# Teratoma Development in 129.MOLF-Chr19 Mice Elicits Two Waves of Immune Cell Infiltration

**DOI:** 10.3390/ijms252312750

**Published:** 2024-11-27

**Authors:** Lucas Klaus, Sybille D. Reichardt, Maria Neif, Lutz Walter, Fabian A. Gayer, Holger M. Reichardt

**Affiliations:** 1 Institute for Cellular and Molecular Immunology, University Medical Center Göttingen, 37073 Göttingen, Germany; lucas.klaus1@med.uni-goettingen.de (L.K.); sybille.reichardt@med.uni-goettingen.de (S.D.R.); maria.neif@ukmuenster.de (M.N.); fabian.gayer@med.uni-goettingen.de (F.A.G.); 2 Department of Dermatology, University Hospital Münster, 48149 Münster, Germany; 3 German Primate Center, Leibniz Institute for Primate Research, 37077 Göttingen, Germany; walter@dpz.eu; 4 Clinic of Urology, University Medical Center Göttingen, 37075 Göttingen, Germany

**Keywords:** testicular germ cell tumor, teratoma, T cells, macrophages, tumor microenvironment

## Abstract

Teratomas are a highly differentiated type of testicular germ cell tumors (TGCTs), the most common type of solid cancer in young men. Prominent inflammatory infiltrates are a hallmark of TGCTs, although their compositions and dynamics in teratomas remain elusive. Here, we reached out to characterize the infiltrating immune cells and their activation and polarization state by using high-throughput gene expression analysis of 129.MOLF-Chr19 mice that spontaneously develop testicular teratomas. We showed that inconspicuous testes without any apparent alterations in size or morphology can be clustered into three groups based on their expression of stemness and immune genes, supporting a model in which initial oncogenic transformation elicits a first wave of T-cell infiltration. Moderately and severely enlarged tumorous testes then displayed a progressive infiltration with T cells, monocytes/macrophages, and B cells. Importantly, T cells seem to adopt an inactive state caused by an overexpression of immune checkpoint molecules and the polarization of monocytes/macrophages to an anti-inflammatory phenotype. Our findings are supported by the analysis of metabolic gene expression, which unveiled alterations indicative of tumor growth and immune cell infiltration. Collectively, testicular teratomas, at least in mice, are characterized by a diverse inflammatory infiltrate containing T cells that putatively become inactivated, allowing the tumors to further grow. We believe that these findings may provide a rationale for the development of new immunomodulatory therapies for TGCTs.

## 1. Introduction

Testicular germ cell tumors (TGCTs) are the most common malignancy in young men [1]. They account for more than 95% of all testicular tumors and show a rising incidence over the last decades [2]. TGCTs are categorized into different forms based on morphology, ontogeny, and the presence of precursor lesions, designated as *germ cell neoplasia* in situ (GCNIS) [3]. TGCTs having developed in dependency of GCNIS are further classified as type II TGCTs and subdivided regarding their gene expression profile and morphology into seminomas and non-seminomatous TGCTs, with the latter representing a highly heterogenous group of tumors [4,5]. Notably, type II TGCTs are malignant tumors, whereas type I tumors grow independently of GCNIS and can be benign [6]. In both cases, their precursors originate in degenerated primordial germ cells that arise during embryogenesis [7]. Amongst TGCTs, teratomas represent a rare subgroup and can either manifest as malignant type II non-seminomatous tumors or benign type I tumors. Even at an advanced stage, malignant TGCTs are highly curable due to the effectiveness of current platinum-based poly-chemotherapy in combination with radical ablation of the affected testis [8].

TGCTs are characterized by inflammatory infiltrates composed of lymphocytes and monocytes/macrophages [9,10]. Their specific roles in the tumor microenvironment (TME) of individual TGCT subtypes and their contribution to the prognosis and overall survival of patients, however, are obscure. T cells and mature macrophages constitute the majority of infiltrating immune cells in TGCTs [9]. The main task of the CD8^+^ cytotoxic T cells (CTL) is to eradicate tumor cells based on the recognition of *de novo* antigens unknown to the host immune system. Consequently, CTL infiltration is associated with tumor elimination mostly through the release of cytolytic granules containing perforin and granzyme B [11]. Monocytes and macrophages can adopt different phenotypes dependent on the presence of specific activating signals in the TME [12]. Pro-inflammatory monocytes/macrophages differentiate following classical activation; for instance, by interferon-gamma (IFNγ). In contrast, polarization toward an anti-inflammatory phenotype occurs after alternative activation mediated by IL-4 and IL-13 [12]. Glucocorticoids, IL-10, or TGFβ can further induce a deactivated phenotype that is distinct from the other two polarization states. Tumor-associated macrophages (TAMs) best resemble the alternatively activated or deactivated phenotypes. They release immunosuppressive factors, recruit regulatory T (Treg) cells, enhance angiogenesis, fibroblast proliferation, and collagen deposition, and eventually promote tumor growth [13]. The signals governing TAM differentiation and their characteristics, however, are still poorly understood. In particular, their role in TGCTs is largely unknown, with the exception that an increased macrophage gene signature was found to be associated with non-seminomatous tumors [14].

Very little knowledge is available concerning the role of immune cells in teratoma development and progression. One study histologically evaluated testes of 129/Sv mice following the experimental engraftment of a teratoma [15]. They could detect the T cell antigens CD4 and CD44, as well as immunoreactivity for cytokines produced by different types of immune and tumor cells, including IL-2, IL-6, and IL-10. In another model, teratomas were analyzed that had developed from embryonic stem cells grafted into mice of the 129 strain [16]. Genes identified in these teratomas were linked to T cells (*Cd3e*, *Cd4*, *Cd8a*, *Ctla4*) and macrophages (*Adgre1*, *Cd80*, *Cd86*, *H2ab*). Flow cytometry analysis further unveiled the presence of memory T cells, Treg cells, and T helper 17 cells [16]. These data support the crucial role of leukocytes in the TME of teratomas, at least in mice. While no detailed analyses on the immune cell composition of human testicular teratomas have been reported, ovarian teratomas associated with autoimmune encephalitis have been studied recently [17]. These tumors contained a prominent inflammatory infiltrate composed of T and B cells organized in follicular structures that was otherwise not observed in control teratomas [17]. Collectively, these findings entail further efforts to study immune cells in testicular teratomas and to characterize their composition and features.

Cancer cells are well-known to switch from oxidative phosphorylation to glycolysis despite being an inefficient pathway. This phenomenon is called the *Warburg effect* and allows transformed cells to rapidly generate the large amounts of ATP needed for rapid growth [18]. Metabolic reprogramming results in an increased expression of glucose transporters and an elevated production of lactate. Immunohistochemical analysis of TGCT specimens confirmed that GLUT1 was overexpressed in these tumors, while HK2 was concomitantly decreased [19]. Importantly, immune cells that have been activated by foreign antigens or neo-antigens expressed by tumor cells undergo metabolic rewiring as well [20]. These changes allow T cells and monocytes/macrophages to cover their high energy demand linked to the production of cytokines, reactive oxygen species, and cytotoxic molecules. Despite the high similarity of metabolic reprogramming in tumor and immune cells, the key players in this process differ. Tumor cells, for instance, import glucose mainly via GLUT1, whereas immune cells in the TME employ GLUT3 for this purpose [21]. Moreover, glycolytic enzymes are differently expressed in both compartments, too. It is against this background that profiling of the metabolic gene signature is believed to be an independent predictor of tumor progression [21].

The consomic mouse strain 129.MOLF-Chr19 was generated in 1957 by introgressing chromosome 19 from the MOLF/Ei strain into the wildtype 129S1/SvImJ background [22]. These mice, also designated M19, develop testicular teratomas with an incidence of up to 80%, which are characterized by the presence of various types of tissues derived from all three germ layers [23,24]. It has been hypothesized that polymorphisms of genes including *Kitl*, *Dmrt1*, *Sf1*, and *Pten* are responsible for tumorigenesis in M19 mice [25,26]. Tumors arise either during embryonic development or after puberty and are reminiscent of human teratomas [27]. We have recently reported the characterization of testes and teratomas in a large cohort of M19 mice and could confirm that their neoplasia contained a variety of tissue differentiation, including muscle, adipose tissue, and cartilage [28]. Gene expression analysis unveiled high levels of stemness markers such as *Sox2* and *Nanog* and proliferation-associated genes like *Myc*. Moreover, expression of these genes was positively correlated with size and suspicion of tumorigenesis. Therefore, we concluded that teratomas in M19 mice are a suitable tool with which to investigate this tumor entity as a model of the human disease.

## 2. Results

### 2.1. Immune Cell Infiltration and Inflammation in Murine Teratomas

**Figure 1:**
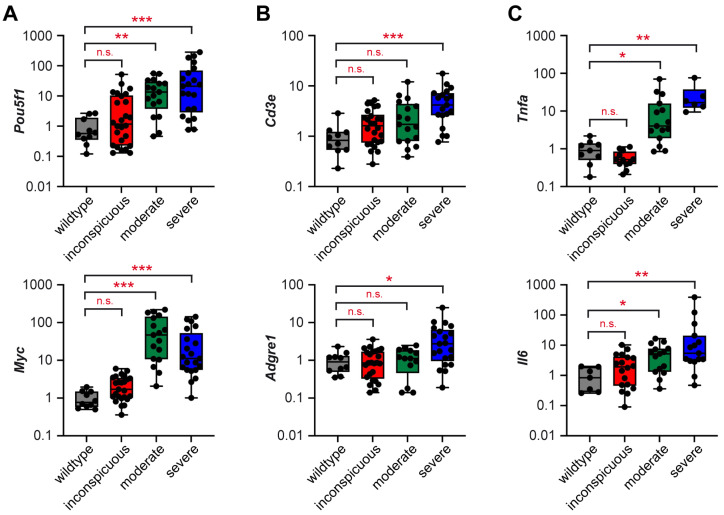
Expression of genes related to neoplastic transformation and immunity in M19 testes at different stages of tumorigenesis. Gene expression of (**A**) *Pou5f1* and *Myc*, (**B**) *Cd3e* and *Adgre1*, and (**C**) *Tnfa* and *Il6* were determined in testes of 129Sv wildtype mice and the three groups of M19 mice using high-throughput Fluidigm^®^ Dynamic Arrays and RT-qPCR (N = 10/26/17/21). Expression of the housekeeping gene *Rn18s* was used for normalization, and mRNA levels in wildtype testes were set to 1. All values are depicted as scatter dot plots with bars representing the mean ± SEM; each dot corresponds to an individual testis. Statistical analysis was performed using a Kruskal–Wallis test followed by Dunn’s multiple comparison test. Levels of significance: *: *p* < 0.05; **: *p* < 0.01; ***: *p* < 0.001; n.s.: *p* > 0.05.

We previously characterized the development of teratomas in testes of M19 mice and subdivided them into three groups based on their size and morphological appearance [28]. Testes were considered inconspicuous if they were similar in size to 129Sv wildtype mice and without any apparent morphological abnormalities. A second group of testes was found to be moderately enlarged, often accompanied by anomalies presumably related to tumorigenesis. Lastly, we observed a number of severely enlarged tumorous testes, characterized by strong purple-red discoloration and cysts. In an expanded cohort of M19 mice, we could here confirm our earlier finding that expression of *Pou5f1* (*Oct3/4)* and *Myc* were significantly enhanced in both groups of overt tumors, which distinguish themselves through their increased size and morphological alterations as described above (Figure 1A). Exemplary photographs of such teratomas have been published previously [28].

The development of TGCTs is generally accompanied by immune cell infiltration and the establishment of a pro-inflammatory TME. To address this issue in teratogenesis, we analyzed gene expression of *Cd3e* and *Adgre1* (*F4/80)* as surrogate markers of T cells and monocytes/macrophages in M19 mice (Figure 1B). *Cd3e* expression was progressively upregulated in testes of M19 mice with increasing suspicion of tumorigenesis, although significance was reached only in strongly enlarged tumors. Changes in *Adgre1* were less pronounced. However, in the severe testicular tumors, a slight but significant increase was detected, too. In addition, a pro-inflammatory TME was observed in both groups of overt tumorous testes, as indicated by strongly enhanced expression of the two key cytokines *Tnfa* and *Il6* (Figure 1C). 

Collectively, our data unveil that the progressive deregulation of genes related to neoplastic transformation is accompanied by an increased abundance of immune cells, in particular T cells, in testes of M19 mice, and the establishment of a pro-inflammatory TME characterized by a prominent expression of cytokines.

### 2.2. Neoplastic Transformation Elicits a First Wave of T-Cell Infiltration

**Figure 2:**
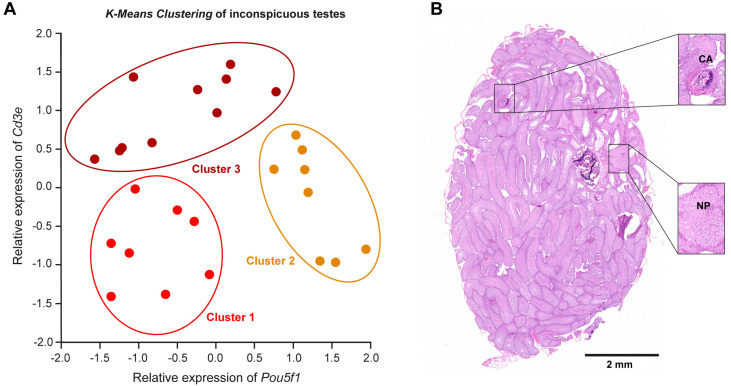
Bioinformatic and histological analysis of inconspicuous testes of M19 mice. (**A**) *K-Means Clustering* of inconspicuous testes into three groups (Clusters 1–3) was achieved based on the relative expression levels of *Pou5f1* and *Cd3e* as determined by using high-throughput Fluidigm^®^ Dynamic Arrays and RT-qPCR (N = 26). Z-Score normalization was used to calculate the relative expression of *Pou5f1* and *Cd3e*. (**B**) Overview and magnifications of an H&E-stained section of an exemplified inconspicuous testis from an M19 mouse (N = 3). Small areas representing tissue differentiations like cartilage (CA) and neuroparenchyma-like tissue (NP) distributed amongst healthy seminiferous tubules with maturing spermatogenesis are depicted in the inlets. The scale bar (2 mm) refers to the overview microphotograph.

Our previous analysis revealed a strong heterogeneity of *Pou5f1* and *Cd3e* expression in inconspicuous testes of M19 mice, which prompted us to perform an unsupervised *K-Means clustering* of these samples based on the expression level of the two genes. Using this machine learning algorithm, we could group inconspicuous testes into three different clusters (Figure 2A). Testes in “Cluster 1” can be described as *Pou5f1*^low^*Cd3e*^low^, showing neither signs of neoplastic transformation nor an increased presence of T cells. In contrast, *Pou5f1* was upregulated in testes falling into “Cluster 2”, while *Cd3e* expression remained low in these specimens. Lastly, testes in “Cluster 3” were characterized by an enhanced expression of the T cell signature gene *Cd3e* accompanied by a partial decline of *Pou5f1*. From these findings, we conclude that some of the inconspicuous testes in M19 mice are indeed devoid of transformed cells. Oncogenic events result in the appearance of tumor cells characterized by increased expression of the stemness marker *Pou5f1*. Consequently, T cells become activated, presumably resulting in partial eradication of the transformed cells expressing *Pou5f1*.

To provide further support for our model, a histological analysis of inconspicuous testes from M19 mice by H&E staining was performed (Figure 2B). Despite their unaltered size and morphological appearance, small clusters of cells representing a variety of tissue differentiations could be detected in several specimens. The observed cell types originate in different germ layers, representing, for instance, cartilage and neuroparenchyma-like tissue (Figure 2B). This finding highlights that the onset of tumorigenesis can be detected by gene expression analysis before it becomes morphologically evident. 

### 2.3. Gene Expression Signature of Inconspicuous Testes in M19 Mice

**Figure 3:**
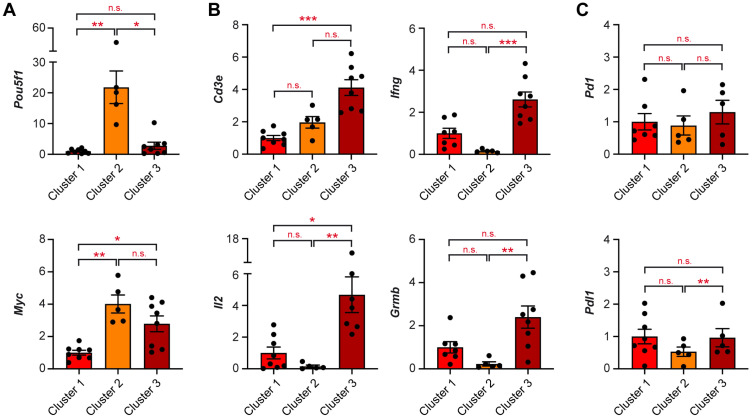
Expression of genes related to tumorigenesis and T cell function in inconspicuous testes of M19 mice. Expression levels of (**A**) *Pou5f1* and *Myc*, (**B**) *Cd3e, Il2, Ifng,* and *Grmb*, and (**C**) *Pd1* and *Pdl1* were determined in the three clusters of inconspicuous testes by high-throughput Fluidigm^®^ Dynamic Arrays and RT-qPCR (N = 5–8). *Rn18s* expression was employed for normalization, and mRNA levels in testes of “Cluster 1” were set to 1. All values are depicted as scatter dot plots with bars representing the mean ± SEM; each dot corresponds to an individual testis. Statistical analysis was performed using a Kruskal–Wallis test followed by Dunn’s multiple comparison test. Levels of significance: *: *p* < 0.05; **: *p* < 0.01; ***: *p* < 0.001; n.s.: *p* > 0.05.

Our analysis unveiled that inconspicuous testes in M19 mice were composed of three different phenotypes. To characterize them further, we initially analyzed gene expression of *Pou5f1* and *Myc.* As expected, both genes were expressed at low levels in “Cluster 1”, strongly increased in “Cluster 2”, and diminished again in “Cluster 3”, albeit to a different degree (Figure 3A).

Next, we studied genes reflecting the abundance and activation state of T cells. *Cd3e* was selectively increased in “Cluster 3” as expected and thus showed an opposite pattern of regulation as compared to *Pou5f1* and *Myc*. Importantly, the T cell effector molecules *Il2*, *Ifng*, and *Grzb* were also significantly induced in “Cluster 3” (Figure 3B). Our findings suggest that this subgroup of testes was not only characterized by a presumably higher abundance of T cells but that they displayed an activated phenotype, too. In contrast, we found that expression of *Pd1* and *Pdl1*, two genes encoding immune checkpoint molecules involved in the control of T cell activity, were not significantly altered in inconspicuous M19 testes (Figure 3C). Nonetheless, the slight trend toward reduced *Pdl1* expression in “Cluster 2” could still indicate that tumor cells at this stage might be unable to suppress the first wave of infiltrating T cells.

**Figure 4:**
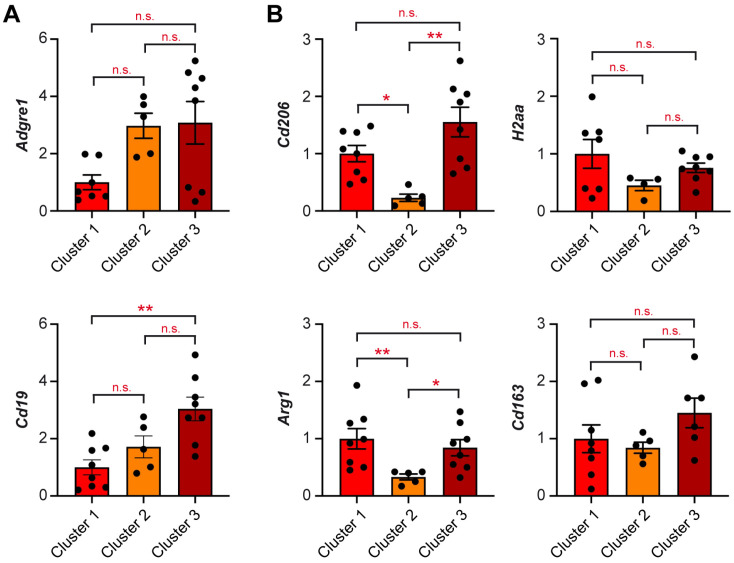
Expression of genes related to monocytes/macrophages and B cells in inconspicuous testes of M19 mice. Expression levels of (**A**) *Adgre1* and *Cd19*, and (**B**) *Cd206*, *Arg1*, *H2aa*, and *Cd163* were determined in the three clusters of inconspicuous testes by high-throughput Fluidigm^®^ Dynamic Arrays and RT-qPCR (N = 5–8). *Rn18s* expression was used for normalization, and mRNA levels in testes of “Cluster 1” were set to 1. Values are depicted as scatter dot plots with bars representing the mean ± SEM; each dot corresponds to an individual testis. Statistical analysis was performed using a Kruskal–Wallis test followed by Dunn’s multiple comparison test. Levels of significance: *: *p* < 0.05; **: *p* < 0.01; n.s.: *p* > 0.05.

Lastly, we reached out to determine the expression of genes related to other immune cells. *Adgre1*, which reflects the abundance of monocytes/macrophages, showed a trend toward higher expression in “Cluster 2” and “Cluster 3”, albeit not reaching significance (Figure 4A). *Cd19* expression was significantly upregulated in “Cluster 3”, suggesting that B cells were enriched in this subgroup of testes, too (Figure 4A). Analysis of *Cd206* and *Arg1*, two genes indicative of an alternatively activated phenotype of monocytes/macrophages, showed they were downregulated in “Cluster 2” (Figure 4B). In contrast, expression of *H2aa* and *Cd163* was largely similar in all inconspicuous testis samples (Figure 4B). These findings indicate that immune cell subtypes are differently affected at the early stage of teratogenesis.

### 2.4. Immune Gene Signature of Overt M19 Teratomas

**Figure 5:**
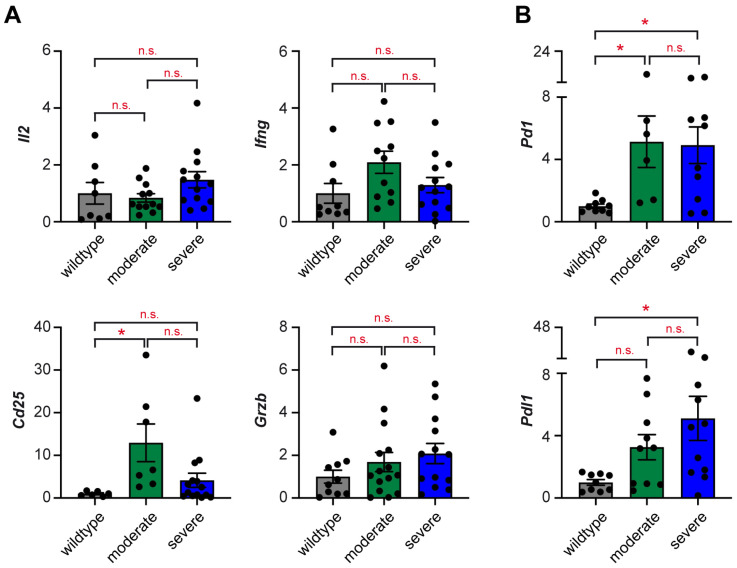
T cell gene signature of overt teratomas in M19 mice. Expression levels of (**A**) *Il2*, *Cd25*, *Ifng*, and *Grzb*, and (**B**) *Pd1* and *Pdl1* were determined in testes of 129Sv wildtype mice and M19 testes with moderate or severe signs of teratogenesis using high-throughput Fluidigm^®^ Dynamic Arrays and RT-qPCR (N = 6–17). *Rn18s* expression was used for normalization, and mRNA levels in wildtype testes were set to 1. Values are depicted as scatter dot plots with bars representing the mean ± SEM; each dot corresponds to an individual testis. Statistical analysis was performed using a Kruskal–Wallis test followed by Dunn’s multiple comparison test. Levels of significance: *: *p* < 0.05; n.s.: *p* > 0.05.

In the majority of M19 mice, the testes show clear signs of tumorigenesis, including a moderately to severely increased size as well as morphological alterations, such as a strong purple-red discoloration and cysts [28]. Our gene expression analyses further indicated that these tumors were characterized by T cell and monocyte/macrophage infiltration, as well as a pro-inflammatory TME (Figure 1B,C). Here, we aimed to further characterize these immune cells in the two groups of overt teratomas of M19 mice. Initially, we studied genes predominantly expressed by activated T cells (Figure 5A). Importantly, we did not observe any differences concerning the T cell effector molecules *Il2*, *Ifng*, and *Grzb* despite the apparent accumulation of T cells in tumors as suggested by the elevated *Cd3e* levels. In contrast, *Cd25* was upregulated, especially in moderately enlarged tumors (Figure 5A). It is noteworthy that this gene is also expressed by Treg cells, which are frequently found in the TME. Interestingly, expression of the two immune checkpoint molecules *Pd1* and *Pdl1* was markedly enhanced in teratomas of M19 mice, suggesting that infiltrating T cells in the tumor become silenced through signaling via the PD-1/PD-L1 axis (Figure 5B).

**Figure 6:**
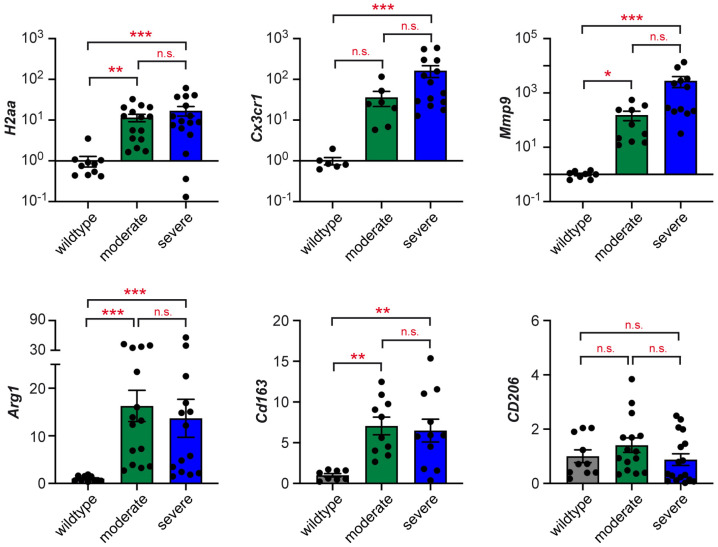
Monocyte/macrophage gene signature of overt teratomas in M19 mice. Expression levels of *H2aa*, *Cx3cr1*, *Mmp9*, *Arg1*, *Cd163*, and *Cd206* were determined in testes of 129Sv wildtype mice and M19 testes with moderate or severe signs of teratogenesis using high-throughput Fluidigm^®^ Dynamic Arrays and RT-qPCR (N = 6–17). *Rn18s* expression was used for normalization, and mRNA levels in wildtype testes were set to 1. Values are depicted as scatter dot plots with bars representing the mean ± SEM; each dot corresponds to an individual testis. Statistical analysis was performed using a Kruskal–Wallis test followed by Dunn’s multiple comparison test. Levels of significance: *: *p* < 0.05; **: *p* < 0.01; ***: *p* < 0.001; n.s.: *p* > 0.05.

Subsequently, we analyzed genes expressed by monocytes/macrophages. The MHC class II gene *H2aa*, the chemokine receptor *Cx3cr1* gene, and the *Mmp9* gene, involved in tissue remodeling, were strongly elevated in M19 teratomas regardless of their degree of severity (Figure 6). To determine the polarization of myeloid cells in the TME, we analyzed *Arg1*, *Cd163*, and *Cd206* expression. While the levels of the former two genes were strongly elevated, *Cd206* expression was unaltered. 

### 2.5. Visualization of T-Cell Infiltration into Teratomas of M19 Mice

**Figure 7:**
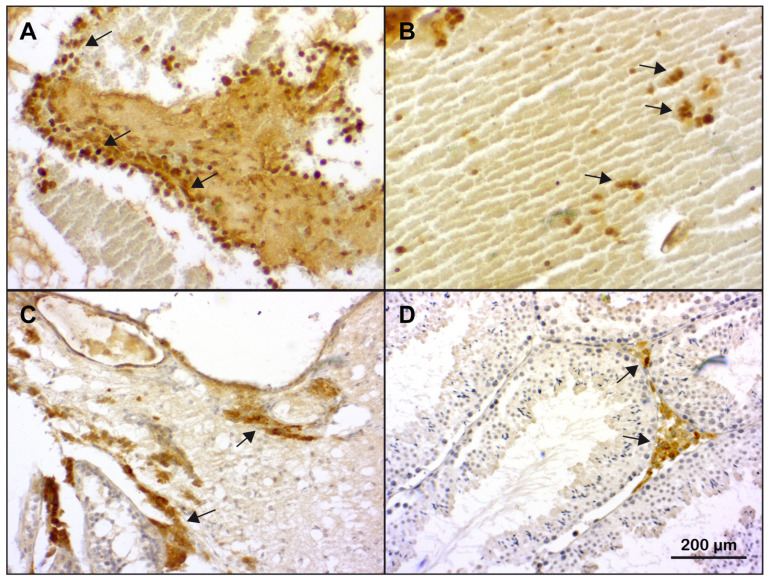
Immunohistochemical analysis of T cells. (**A**–**C**) T cells were visualized in paraffin sections prepared from an exemplary overt teratoma of M19 mice (N = 3) using a primary anti-CD3 antibody in combination with a polymeric secondary antibody, DAB as a substrate, and hematoxylin for counterstaining. Representative photomicrographs of tissue differentations found in the teratoma acquired at 20× magnification are depicted. (**A**) T cells were located at the border of tumerous tissue, (**B**) in differentiated skeletal muscle, and (**C**) neuroparenchyma-like tissue (indicated by arrows). (**D**) T cells were visualized in paraffin sections of a testis obtained from a 129Sv wildtype mouse (N = 3) and located in the interstitial space (indicated by arrows). Positive and negative staining controls are depicted in Supplementary Figure S1. Scale bar = 200 µm in all panels.

Our gene expression analyses indicated that teratomas were strongly infiltrated by T cells. To obtain additional direct evidence of the presence of T cells in the tumors of M19 mice, we performed an immunohistochemical analysis of three exemplary teratomas of M19 mice classified as severely enlarged with purple-red discoloration and cysts. Testes from 129Sv wildtype mice served as controls. Foci of abundant T cells were identified at the border of tumorous tissue (Figure 7A). Moreover, T cells were also present in differentiated skeletal muscle and neuroparenchyma-like tissue (Figure 7B,C). It is noteworthy that T cells were identified in all tumor specimens, although the staining pattern differed between individual teratomas due to the diversity of tissue differentiations found in each of them [28]. In wildtype testes, T cells were located in the interstitial space as expected (Figure 7D). Collectively, our findings confirm that tumors in M19 mice are indeed infiltrated by T cells, as already suggested by our gene expression analyses.

### 2.6. Changes in the Glycolytic Profile of Teratomas in M19 Mice

Tumor cells are characterized by profound changes in the metabolic gene signature, which involves a switch from oxidative phosphorylation to glycolysis [18]. Importantly, a similar rewiring of the energy metabolism occurs in T cells and monocytes/macrophages after activation [20]. The genes involved in glucose transport and glycolysis in both cell types, however, appear to be different, thus representing promising surrogate candidates for the tumor immune profile [21]. It is against this background that we tested expression of several of these genes at different stages of teratogenesis in testes of M19 mice.

**Figure 8:**
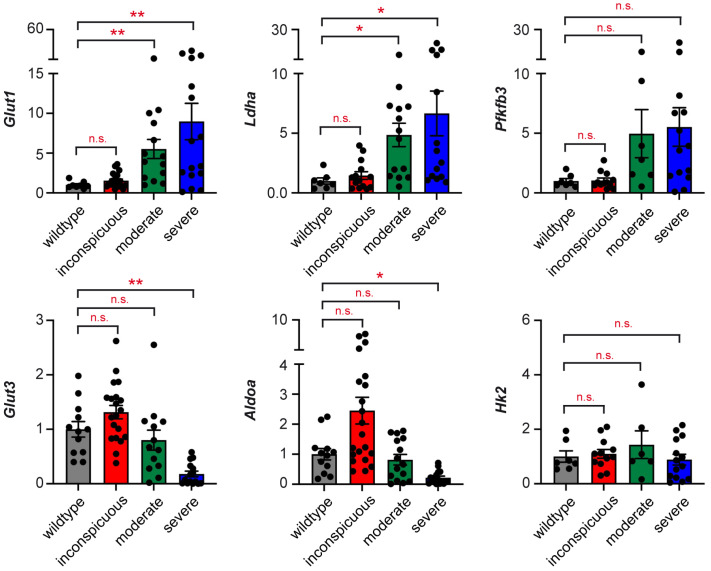
Metabolic gene signature of M19 testes at different stages of teratogenesis. Expression levels of *Glut1*, *Ldha*, *Pfkfb3*, *Glut3*, *Aldoa*, and *Hk2* were determined in testes of 129Sv wildtype mice, inconspicuous testes of M19 mice, and M19 testes with moderate or severe signs of teratogenesis by high-throughput Fluidigm^®^ Dynamic Arrays and RT-qPCR (N = 7–21). *Rn18s* expression was used for normalization, and mRNA levels in wildtype testes were set to 1. Values are depicted as scatter dot plots with bars representing the mean ± SEM; each dot corresponds to an individual testis. Statistical analysis was performed using a Kruskal–Wallis test followed by Dunn’s multiple comparison test. Levels of significance: *: *p* < 0.05; **: *p* < 0.01; n.s.: *p* > 0.05.

*Glut1*, *Ldha*, and *Pfkfb3* were strongly elevated in moderately and severely tumorous testes (Figure 8). In contrast, *Glut3* and *Aldoa* were downregulated in the most advanced stage of teratogenesis. Surprisingly, we did not detect any changes in *Hk2* expression (Figure 8). The observed gene expression signature is compatible with tumor progression and a concomitant silencing of immune cells in advanced stages of teratogenesis. Thus, the metabolic gene signature reflects oncogenic transformation and immune cell activity in murine testicular tumors.

### 2.7. K-Means Clustering of M19 Tumor Samples Based on Gene Expression

**Figure 9:**
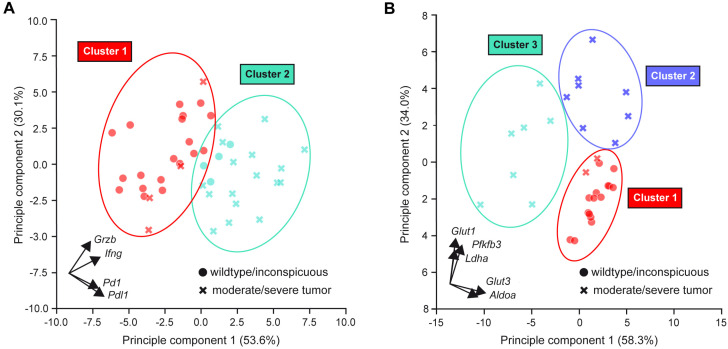
PCA followed by *K-Means clustering* of gene expression during teratogenesis in M19 mice. (**A**) Clustering of testis samples based on *Ifng*, *Grzb*, *Pd1*, and *Pdl1* expression. (**B**) Clustering of testis samples based on *Glut1*, *Ldha*, *Pfkfb3*, *Glut3*, and *Aldoa* expression. Z-Score normalization was used to calculate relative expression levels. The colors refer to the two/three separate clusters that were identified by unsupervised *K-Means clustering*. Testes from 129Sv wildtype mice and inconspicuous testes of M19 mice are displayed as dots, and moderately and severely tumorous testes from M19 mice are displayed as cross marks. The vectors in the inserts indicate the contribution of each analyzed gene to both principal components.

Our expression analyses revealed that genes related to T cell effector functions on the one hand and glycolytic activity on the other hand appear to have strong predictive value for the presence of a testicular teratoma in M19 mice. To support this conclusion, we performed a principal component analysis (PCA) for dimensionality reduction with a representative set of genes, followed by unsupervised *K-Means clustering*. The differential expression of the T cell effector molecules *Ifng* and *Grzb* and the immune checkpoint molecules *Pd1* and *Pdl1* resulted in one cluster mostly composed of wildtype and inconspicuous testes and a second cluster composed of moderately and severely tumorous testes (Figure 9A). Using the same strategy, three clearly separated clusters were identified based on the metabolic genes *Glut1*, *Ldha, Pfkfb3, Glut3,* and *Aldoa* (Figure 9B). The latter clustering suggests a model in which tumors initially overexpress *Glut1*, *Ldha,* and *Pfkfb3* (“Cluster 2”), implying teratoma growth, which is then followed by a downregulation of *Glut3* and *Aldoa* (“Cluster 3”), putatively indicating immune cell suppression. Altogether, the gene signature of teratomas in M19 mice provides valuable insights into the role of the immune response in tumor progression.

## 3. Discussion

TGCTs are a malignancy that predominantly occurs in young men. Although they are well curable, metastasis and chemoresistance still pose considerable problems, entailing further efforts to characterize this tumor entity [2]. Here, we set out to study how the immune system interacts with testicular teratomas by performing an extensive expression profiling of genes related to immune cell function and metabolism using specimens collected from M19 mice. This murine model spontaneously develops tumors closely resembling human testicular teratomas [28]. While the analysis of an expanded sample set confirmed our recently published finding that tumors overexpress the pluripotency- and proliferation-associated genes *Pou5f1* and *Myc*, we now provide data demonstrating how teratomas in M19 mice are infiltrated by T cells, monocytes/macrophages, and B cells.

TGCTs in humans are generally characterized by a pro-inflammatory TME, which is most pronounced in seminomas but also found in teratomas [29]. Here, we observed that *Il6* and *Tnfa* expression was increased in moderately and severely enlarged tumors in M19 mice. In healthy testes, IL-6 is secreted by Leydig and Sertoli cells [30], whereas TNFα is produced by spermatids [31]. Furthermore, expression of these cytokines was found to be significantly increased in seminomas [10]. Our results now suggest that these cytokines are also excessively expressed in our tumor model, thus creating a similar pro-inflammatory TME in murine teratomas. IL-6 and TNF-α have been associated with a disruption of the blood-testis-barrier (BTB), which normally protects germ cells from autoreactive immune cells [32,33]. It is thus reasonable to assume that the BTB also prevents neoplastic germ cells from being recognized by T cells and that disruption of the BTB by IL-6 and TNF-α might therefore be crucial for eliminating neoplastic germ cells by infiltrating immune cells.

The testes in a subgroup of M19 mice are similar in size to 129Sv wildtype mice and lack any signs of tumorigenesis. Unsupervised *K-Means clustering* now revealed that these inconspicuous testes can be subdivided into three separate clusters. One group displayed low expression of *Pou5f1* and *Cd3e* and was thus most similar to 129Sv wildtype samples. Another group displayed high *Pou5f1* and low to moderate *Cd3e* levels. We reasoned that overexpression of *Pou5f1* indicates the presence of transformed OCT4^+^ EC-like germ cells and initial infiltrating T cells. It appears that the immune response at this stage prevents the transformed EC-like germ cells from extensively growing, albeit being unable to fully eliminate the neoplastic cells. The last group is then characterized by elevated *Cd3e* levels, whereas *Pou5f1* expression is at most moderately increased. It is tempting to speculate that a transformation of germ cells has taken place in these particular testes, although infiltrating CTL had been able to eliminate the majority of this OCT4^+^ EC-like germ cell population. This model is supported by our histological finding that individual inconspicuous tumor specimens contain small areas consisting, for instance, of neuroparenchyma-like tissue or cartilage, qualifying them as teratomas. We conclude that an initial phase of tumorigenesis with few transformation events is followed by T-cell infiltration. If the balance between tumor and immune system becomes tilted to the former one, the neoplastic cells continue to proliferate and develop into overt teratomas.

Gene expression analysis of the inconspicuous testes provided us with the unique opportunity to shed light onto the early events of teratoma development. When analyzing testis samples from “Cluster 3”, we did not only note an increased abundance of T cells based on *Cd3e* expression but also higher levels of T cell effector molecules. *Il2* expression reflects the proliferative activity of T cells, *Ifng* is a cytokine predominantly expressed by CTL, and *Grzb* is involved in apoptosis. Hence, overexpression of these genes is testimony to the induction of an immune response that is dominated by CTL, presumably directed against the neoplastic cells. We could also show an increased abundance of B cells as well as monocytes/macrophages in testes falling into “Cluster 3” based on *Adgre1* and *Cd19* expression. Surprisingly, the myeloid cells seemingly adopt an unconventional phenotype because they highly express *Cd206* and *Arg1,* which are typically related to alternatively activated or deactivated macrophages. In contrast, the MHC class II gene *H2aa* and the scavenger receptor *Cd163* were unaltered. These findings entail a model in which T cells attack the tumor cells, which conversely promote polarization of monocytes/macrophages toward an immunosuppressive phenotype.

The majority of testes in M19 mice are enlarged and tumorous, as described recently [28]. Despite the high abundance of T cells in these teratomas, as suggested by our gene expression and immunohistochemical analyses, they appear to be functionally silenced. This conclusion is justified by our finding that genes encoding effector molecules, such as *Il2*, *Ifng,* and *Grzb*, were unaltered in moderately and severely enlarged tumors, whereas genes encoding the immune checkpoint molecules *Pd1* and *Pdl1* were induced. It is known that signaling via the PD-1/PD-L1 axis can inhibit tumor-infiltrating CTL by diminishing cytokine production and inducing T cell apoptosis [34]. Expression of PD-L1 has been described in different types of TGCTs, albeit to a lesser extent in teratomas as compared to other histological subtypes [35]. The high expression of *Cd25*, in the group of moderately enlarged tumors in particular, could contribute to the immunosuppressive TME in M19 teratomas, too. CD25 is constitutively expressed by Treg cells [36], and their progressive infiltration has been observed in non-seminomas, including teratomas [14]. Monocytes and macrophages are also abundantly found in the advanced stages of M19 tumors, as suggested by the elevated expression of *H2aa*, *Cx3cr1,* and *Cd163*. Interestingly, these cells have adopted an alternatively activated phenotype based on their high expression of *Arg1*. Nonetheless, they are still unconventional in the sense that *Cd206* levels are not altered. Taken together, T cells in the TME of advanced stages of teratomas seem to be in a suppressed state due to the presumed signaling via the PD-1/PD-L1 axis and the presence of Treg cells and TAMs.

A growing tumor not only needs to suppress the immune response but also has to break through the seminiferous tubules. A recent study demonstrated co-localization of the macrophage marker CD68 and the metalloproteinase MMP9 in seminomas [37]. We observed a significantly higher expression of *Mmp9* in moderately and severely enlarged M19 tumor samples, too, indicating a similar role of MMP9 in teratomas. Although we did not notice any signs of malignancy in our M19 mice up to now [28], it is worth mentioning that spontaneous metastases with putative germ cell origin were previously observed in the 129Sv family of mice by others [38]. 

Metabolic reprogramming is a typical feature of tumors. Originally described by Otto Warburg, neoplastic cells switch to glycolysis to support their rapid proliferation [18]. More recently, it was found that immune cells also change their metabolic profile upon activation, thereby promoting their differentiation into effector cells [20]. It is against this background that we tested the expression of glucose transporters and glycolytic enzymes in teratomas from M19 mice. *Glut1*, *Ldha*, and *Pfkfb3* were overexpressed in moderately to severely enlarged tumors, while *Glut3* and *Aldoa* displayed a significant downregulation in the most advanced tumor stage. Deregulation of GLUT1 [39], PFKFB3 [40], and LDHA [41] is known in many forms of cancers and facilitates the high glucose consumption by tumor cells. CTL and macrophages, however, preferentially use GLUT3 instead of GLUT1 for glucose import [42]. Hence, our data suggest that the metabolic gene signature is an independent predictor of the immune profile of teratomas. 

Bioinformatic analysis of gene expression data is a powerful tool to obtain insights into the features of tumor samples. We used unsupervised *K-Means clustering* based on the expression of T cell-related and metabolic genes. Hereby, we were able to demonstrate that clustering based on the expression profile matches the testicular phenotype, namely wildtype and inconspicuous testes on the one hand and moderately and severely enlarged tumorous testes on the other hand, at least in the vast majority of cases. The slight overlap between individual clusters supports the view that teratogenesis is a continuous process that proceeds from inconspicuous testes with initial neoplastic transformation and the upset of immune cell infiltration to overt tumors.

Up to now, our observations with regard to the immunological features of testicular teratomas are limited to mice. Hence, it will be important in the future to investigate whether these findings can be translated to human pathology as well. Since no cell lines derived from testicular teratomas are available, we will depend on the analysis of patient specimens in this respect. We have already collected blood and tissue samples from more than 80 TGCT patients, but only around 5% of them represent pure teratomas. Therefore, additional efforts are needed before a detailed analysis of the immune infiltrate in human teratomas can be provided. 

## 4. Materials and Methods

### 4.1. Animal Experimentation

129S1/SvImJ-Chr19^MOLF/Ei^/NadJaheJ mice (strain number: 029319), designated M19, were described previously [28] and bred in our animal facility at the University Medical Center Göttingen. Due to the transfer of chromosome 19 from MOLF/Ei to 129S1/SvImJ mice, the incidence of spontaneous teratoma development increased from approximately 5% to around 80%. All animal experiments were approved by the responsible authorities (*Nds. Landesamt für Lebensmittelsicherheit und Verbraucherschutz*) and comply with ethical standards of animal welfare and the ARRIVE guidelines. Testes or testicular tumors were collected from male mice sacrificed at different ages by using CO_2_ inhalation followed by cervical dislocation. Some specimens have been previously used for basic characterization of tumor-associated genes [28], whereas others are reported here for the first time.

### 4.2. Quantitative RT-PCR Analysis

Tissue biopsies were snap-frozen in dry ice and stored at −80°C for further processing. Specimens were first homogenized using an Ultra Turrax T18 device (IKA Laboratory Technology, Staufen, Germany), followed by RNA isolation employing the RNeasy Mini Kit (Qiagen, Hilden, Germany). Total RNA was reverse transcribed into cDNA with the help of the iScript Kit (Bio-Rad, Munich, Germany). RT-qPCR was performed on an ABI 7500 instrument using a SYBR green master mix (both from ThermoFisher, Darmstadt, Germany). The housekeeping gene *Rn18s* was employed for normalization, and relative levels of gene expression were determined with the ΔΔCt method. All primers were synthesized by Metabion (Planegg, Germany); the sequences are listed in Supplementary Table S1. 

### 4.3. High-Throughput Fluidigm^®^ Dynamic Arrays

To perform a concomitant analysis of gene expression of a large number of samples with a set of 24 primers, 48.48 Dynamic Arrays (Fluidigm^®^, San Francisco, CA, USA) and the BioMark HD system were employed as previously described [28,43]. The BioMark Data Collection software (Fluidigm; version 4.8.2) was employed for high-throughput RT-qPCR and melting curve analysis. The housekeeping gene *Rn18s* was used for normalization, and relative expression levels were calculated by using the ΔΔCt method.

### 4.4. Histology and Immunohistochemistry

Testis specimens were fixed in 4% Roti-Histofix (Carl Roth, Karlsruhe, Germany), dehydrated using ascending concentrations of ethanol, and finally embedded in paraffin. Histopathological evaluation was performed after staining 2 µm sections with hematoxylin and eosin (H&E) according to our standard protocol. Immunohistochemical staining was performed overnight with a primary antibody recognizing CD3 (1:100; Bio-Rad, #MCA1477) following antigen retrieval with 0.1 M citrate buffer. Sites of immunoreactivity were visualized with the help of a polymeric secondary antibody coupled to HRP using the ImmPRESS HRP Polymer Detection Kit (Vector Laboratories, Burlingame, CA, USA) in combination with 3’3’-diaminobenzidine (DAB; Agilent Technologies, Santa Clara, CA, USA). Hematoxylin was used for counterstaining. Microphotographs were acquired with an Axio Scope A1 microscope (Leica, Wetzlar, Germany) or by scanning the slides with a computer-directed microscope stage (Olympus SLIDEVIEW VS200, Evident, Hamburg, Germany).

Histological sections prepared from the spleen of a wildtype C57BL/6 mouse were used as controls (Supplementary Figure S1). Incubation with the anti-CD3 antibody and the polymeric secondary antibody revealed the expected staining pattern of T cells located in splenic follicles (positive control). In contrast, no positively stained cells were found in splenic sections incubated only with the secondary antibody while omitting the primary anti-CD3 antibody (negative control). 

### 4.5. Unsupervised Clustering of Gene Expression Data

Unsupervised *K-Means Clustering* was performed to identify sample clusters based on gene expression using the K-Means module from the Python “scikit-learn” library (version 1.5.1). Gene expression data were log_2_-transformed and standardized by Z-score normalization employing the “StandardScaler” module. For dimensionality reduction, the PCA module from the Python “scikit-learn” library (version 1.5.1) was used. The number of clusters was determined by the sum of squared distances to the cluster centers (“elbow method”). The data were visualized as a scatter plot using the Python “Matplotlib” library (version 3.9.0).

### 4.6. Statistical Analysis

Data were analyzed by using the non-parametric *Kruskal–Wallis* test, followed by Dunn’s multiple comparison test. Analyses were performed with GraphPad Prism^®^ software, version 9 (San Diego, CA, USA). Data are depicted as scatter dot plots or dot-box plots with the mean ± SEM indicated in each graph. Levels of significance: n.s.: *p* > 0.05; *: *p* < 0.05; **: *p* < 0.01; ***: *p* < 0.001.

## 5. Conclusions

Our results demonstrate that immune cells and pro-inflammatory cytokines shape the TME of teratomas in M19 mice. Initial neoplastic transformation events elicit a first immune response. With progressing tumorigenesis, T cells then become inactivated by PD-1/PD-L1 signaling, Treg cells, and TAMs, which eventually allows the teratomas to develop into large tumors. While our findings in M19 mice are highly relevant for the human type I subtype due to their overall similarity [19], we can only speculate whether the identified processes are also comparable to what happens in human type II non-seminomatous teratomas. Thus, further evidence is needed to assess the relevance of our findings for human pathology.

## Data Availability

Data and material are available from the corresponding author on reasonable request.

## Supplementary Materials

The following supporting information can be downloaded at: https://www.mdpi.com/article/10.3390/ijms252312750/s1.
