# Supplementary material for: Teratoma Development in 129.MOLF-Chr19 Mice Elicits Two Waves of Immune Cell Infiltration
Source: Int J Mol Sci. 2024 Nov 27;25(23):12750. doi: 10.3390/ijms252312750 (PMC11640870; doi:10.3390/ijms252312750)
Supplement: Supplementary file 1 [file ijms-25-12750-s001.zip › ijms-3306038-supplementary.pdf]

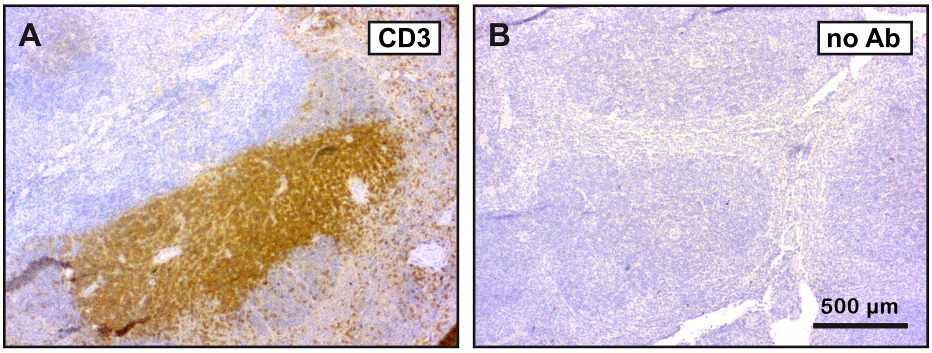

Supplementary Figure S1. Immunohistochemical control analyses. Staining of paraffin sections prepared from the spleen of a C57BL/6 mouse **(A)** with an anti-CD3 antibody and a polymeric secondary antibody (positive control), or **(B)** omitting the primary antibody (negative control). Exemplary photomicrographs acquired at 10x magnification are depicted. Scale bar = 500 µm.

Supplementary Table S1. Primer sequences

Gene	Forward primer	Reverse primer
Adgre1	ACC CTC CAG CAC ATC CAG CC	TCA CAG CCC GAG GGT GTC CA
Aldoa	CAG ATG GGT CCA GCT TCA AC	TGC TTT CCT TTC CTA ACT CTG
Arg1	AGC CCG AGC ACA TGC AGC AG	ACC CCT CCT CGA GGC TGT CCT
Cd163	GCA GTT TCC TCA AGA GGA GAG AA	ATG GCC TCC TTT TCC ATT CCA
Cd19	TCC CTC GGG AAA CGG GAC CC	TGA GCA TGG GCC TGG CCA GA
Cd206	TCC GAA ATG TTG AAG GGA	ATT ATT CCA AAG GCC CGA
Cd25	TGG CAA CAC AGA TGG AGG AA	CGT TAG GTG AAT GCT TGG CG
Cd3e	TGG AAC ACT TTC TGG GGC AT	TCA ATG TTC TCG GCA TCG TCC
Cx3cr1	TGT CCA CCT CCT TCC CTG AA	TCG CCC AAA TAA CAG GCC
Glut1	AGC ATC TTC GAG AAG GCA GG	ACA ACA AAC AGC GAC ACC AC
Glut3	CTC TTC AGG TCA CCC AAC TA CGT	CCG CGT CCT TGA AGA TTC C
Gzmb	TGT GGG CCC CCA AAG TGA CA	AAA GGC AGG GGA GAT CAT CGG G
H2aa	TGA TTC TGG GGG TCC TCG CCC	ACG TGG TCG GCC TCA ATG TCG
Hk2	GCC TCG GTT TCT CTA TTT GGC	ATA CTG GTC AAC CTT CTG CAC T
Ifng	ACT GGC AAA AGG ATG GTG AC	TGA GCT CAT TGA ATG CTT GG
Il2	ACT TGC CCA AGC AGG CCA CA	CCA GAA CAT GCC GCA GAG GT
Il6	AGT TGC CTT CTT GGG ACT GA	CAG AAT TGC CAT TGC ACA AC
Ldha	AGC TTC CAT TTA AGG CCC CG	TCT TTT GAG ACC GCT AGT GC
Mmp9	GGT CTT CCC CAA AGA CCT GAA A	AAT GGC CTT TAG TGT CTG GCT
Myc	TTG GAA ACC CCG CAG ACA G	GCT GTA CGG AGT CGT AGT CG
Pd1	AAC CAG AAG GCC GGT TTC AA	TCC TCT GGC CTC TGA CAT ACT
Pdl1	CGC CTG CAG ATA GTT CCC AA	AGC CGT GAT AGT AAA CGC CC
Pfkfb3	GTC GCC GAA TAC AGC TAC GA	CCC ACA GGA TCT GGG CAA C
Pou5f1	GGA TGG CAT ACT TG GAC CTC	TTT CAT GTC CTG GGA CTC CTC G
Rn18s1	GTA ACC CGT TGA ACC CCA	CCA TCC AAT CGG TAG TAG
Tnfa	ATG GCC TCC CTC TCA TCA GT	CTT GGT GGT TTG CTA CGA CG
